# Identification of Periostin as a Critical Marker of Progression/Reversal of Hypertensive Nephropathy

**DOI:** 10.1371/journal.pone.0031974

**Published:** 2012-03-05

**Authors:** Dominique Guerrot, Jean-Claude Dussaule, Mouna Mael-Ainin, Yi-Chun Xu-Dubois, Eric Rondeau, Christos Chatziantoniou, Sandrine Placier

**Affiliations:** 1 Institut National de la Santé Et de la Recherche Médicale UMRS 702, Hôpital Tenon, Paris, France; 2 Université Pierre et Marie Curie, Paris, France; 3 Service de Néphrologie, Centre Hospitalier-Universitaire Hôpitaux de Rouen, Rouen, France; 4 Service de Physiologie, Hôpital Saint-Antoine, Assistance Publique - Hôpitaux de Paris, Paris, France; 5 Urgences Néphrologiques et Transplantation Rénale, Hôpital Tenon, Assistance Publique - Hôpitaux de Paris, Paris, France; National Center for Scientific Research Demokritos, Greece

## Abstract

Progression of chronic kidney disease (CKD) is a major health issue due to persistent accumulation of extracellular matrix in the injured kidney. However, our current understanding of fibrosis is limited, therapeutic options are lacking, and progressive degradation of renal function prevails in CKD patients. Uncovering novel therapeutic targets is therefore necessary.

We have previously demonstrated reversal of renal fibrosis with losartan in experimental hypertensive nephropathy. Reversal was achieved provided that the drug was administered before late stages of nephropathy, thereby determining a non-return point of CKD progression. In the present study, to identify factors critically involved in the progression of renal fibrosis, we introduced losartan at the non-return point in L-NAME treated Sprague Dawley rats. Our results showed either reversal or progression of renal disease with losartan, defining 2 groups according to the opposite evolution of renal function. We took advantage of these experimental conditions to perform a transcriptomic screening to identify novel factors potentially implicated in the mechanisms of CKD progression. A secondary analysis of selected markers was thereafter performed. Among the targets identified, periostin, an extracellular matrix protein, presented a significant 3.3-fold higher mRNA expression in progression compared to reversal group. Furthermore, independent of blood pressure, periostin was strongly correlated with plasma creatinine, proteinuria and renal blood flow, hallmarks of hypertensive renal disease severity. Periostin staining was predominant in the injured regions, both in experimental hypertensive and human nephropathy.

These results identify periostin as a previously unrecognized marker associated with disease progression and regression in hypertensive nephropathy and suggest measuring periostin may be a sensitive tool to evaluate severity, progression and response to therapy in human kidney disease associated to hypertension.

## Introduction

In the kidney, sustained insult commonly leads to an increased synthesis of extracellular matrix, which surrounds and eventually replaces the injured structures. In chronic kidney diseases, this fibrotic process spontaneously autoaggravates and contributes to a progressive reduction in the number of functioning nephrons, irrespective of the initial cause of the disease [1], [2]. Therefore, understanding mechanisms responsible for the progression or the reversal of fibrosis is a major therapeutic target. Over the past decade, several key contributors to the pathophysiology of fibrosis have been identified, including components of the renin-angiotensin-aldosterone system, transforming growth factor-beta1 (TGF-beta1), regulators of cell plasticity, and proinflammatory cytokines such as monocyte chemoattractant protein-1 (MCP-1) [3], [4]. Recent studies have also underlined the importance of extracellular matrix proteins, not only as components of the fibrotic scar, but also as active regulators of tissue remodeling *via* cell-matrix signaling [5]–[7].

We have previously demonstrated the possibility of therapeutic reversal of renal fibrosis in experimental hypertensive nephropathy, especially with losartan, an angiotensin II receptor antagonist [8]–[10]. Fibrogenesis is a multistep process and therapeutic efficacy requires timely treatment. Specifically, the introduction of losartan beyond a non-return point of experimental renal fibrosis fails to achieve control of the profibrotic mechanisms.

In the present study we hypothesized that actors crucially involved in the orientation of disease at the non-return point may play an important role in the pathophysiology of renal fibrosis, and may consequently be useful biomarkers of ongoing injury and promising therapeutic targets. To identify candidate proteins we performed a transcriptomic analysis of factors associated with the progression of chronic kidney disease. Thereafter, we further characterized selected targets at different stages of hypertensive nephropathy, including progression and reversal of renal disease after introduction of losartan. We concluded that periostin expression more than indices of endothelial or tubular dysfunction was strongly related to the progression and the regression of experimental hypertensive nephropathy, independently of changes in systolic blood pressure.

## Results

### Pharmacological nitric oxide inhibition induces progressive renal vascular disease

**Figure 1:**
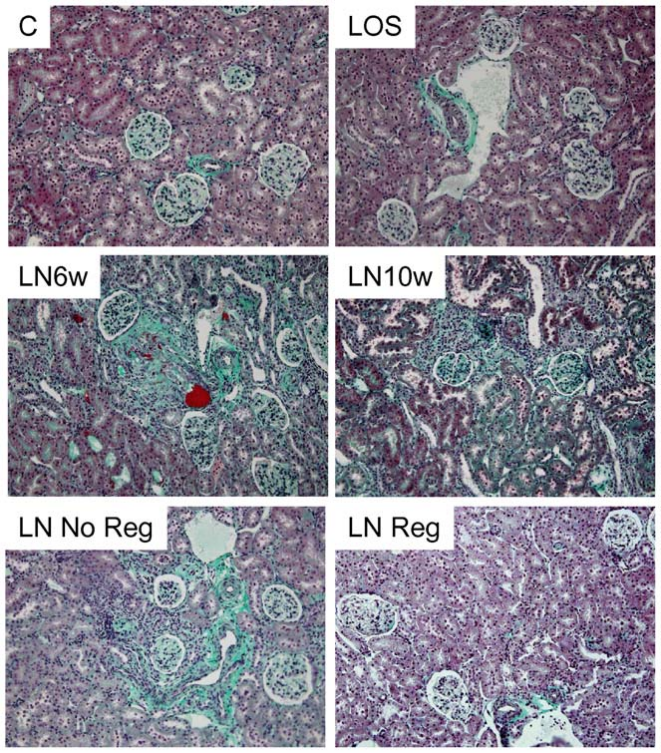
Masson's trichrome staining of renal cortex. Original magnification ×20.

**Figure 2:**
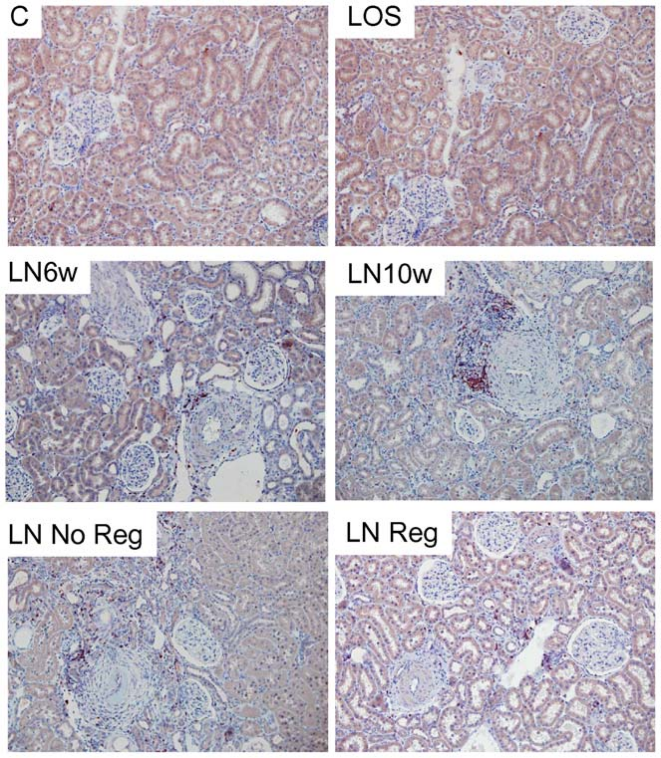
CD3 immunohistochemistry in renal cortex. Original magnification ×20.

After initiation of L-NAME treatment, rats rapidly developed severe persistent hypertension (MAP = 211±7 mmHg, and 212±5 mmHg at 6 and 10 weeks treatment respectively) (Table 1). Progressive hypertensive renal disease was characterized by the early onset of proteinuria (1.3±0.2 g/mmol creatininuria at week 6) and a delayed increase in creatininemia (100±14 µmol/l at week 10). Renal blood flow exhibited a striking reduction from 6 weeks L-NAME treatment onwards. As expected, these functional alterations were associated with progressive histological lesions of vascular nephropathy including glomerulosclerosis, vascular fibrosis, interstitial fibrosis, tubular lesions and inflammation (Figure 1, Table 1). A characterization of the inflammatory infiltrating cells showed that CD3+ lymphocyte count was strongly increased after 6 and 10 weeks L-NAME treatment (Figure 2, Table 1).

**Table 1:** Descriptive statistics for functional and structural parameters of hypertensive nephropathy.

	C	LOS	LN 6w	LN 10w	LN reg	LN no reg
*Functional parameters*						
MAP (mmHg)	128±4	118±9	211±7* #	212±5* #	167±5*	202±5* #
P/C (g/mmol)	0.16±0.03	0.17±0.02	1.31±0.24* #	0.95±0.13* #	0.30±0.02	0.45±0.06*
pCr (µmol/l)	43±2	43±2	64±8	100±14* #	50±2	76±3* #
RBF (ml/min)	10.1±0.3	10.5±0.4	4.4±0.5* #	3.3±0.4* #	8.8±0.4	6.4±0.5* #
*Histological scores*						
Interstitial Fibrosis	0.3±0.2	0.2±0.1	0.6±0.3	2.1±0.4* #	0.7±0.2	1.1±0.2*
Vascular Fibrosis	0.2±0.1	0.2±0.2	0.6±0.2	1.6±0.3* #	0.4±0.2	1.3±0.2* #
Glomerulosclerosis	0.1±0.1	0.2±0.3	0.6±0.2	1.4±0.4* #	0.3±0.1	1.0±0.2* #
Tubular Lesions	0.1±0.1	0.1±0.2	0.5±0.2	1.8±0.4* #	0.4±0.1	0.8±0.2*
Inflammation	0.4±0.2	0.3±0.3	1.4±0.5* #	2.2±0.4* #	0.6±0.1	1.9±0.2* #
CD3+ cells/mm^2^	6±1	6±2	53±6*	100±14* #	37±6*	67±12* #

C: Control, LOS: Losartan, LN: L-NAME, LN Reg: L-NAME Regression, LN No Reg: L-NAME No Regression.

MAP: Mean Arterial Pressure, P/C: Proteinuria/Creatininuria, pCr: Plasma Creatinine, RBF: Renal Blood Flow.

**Figure 3:**
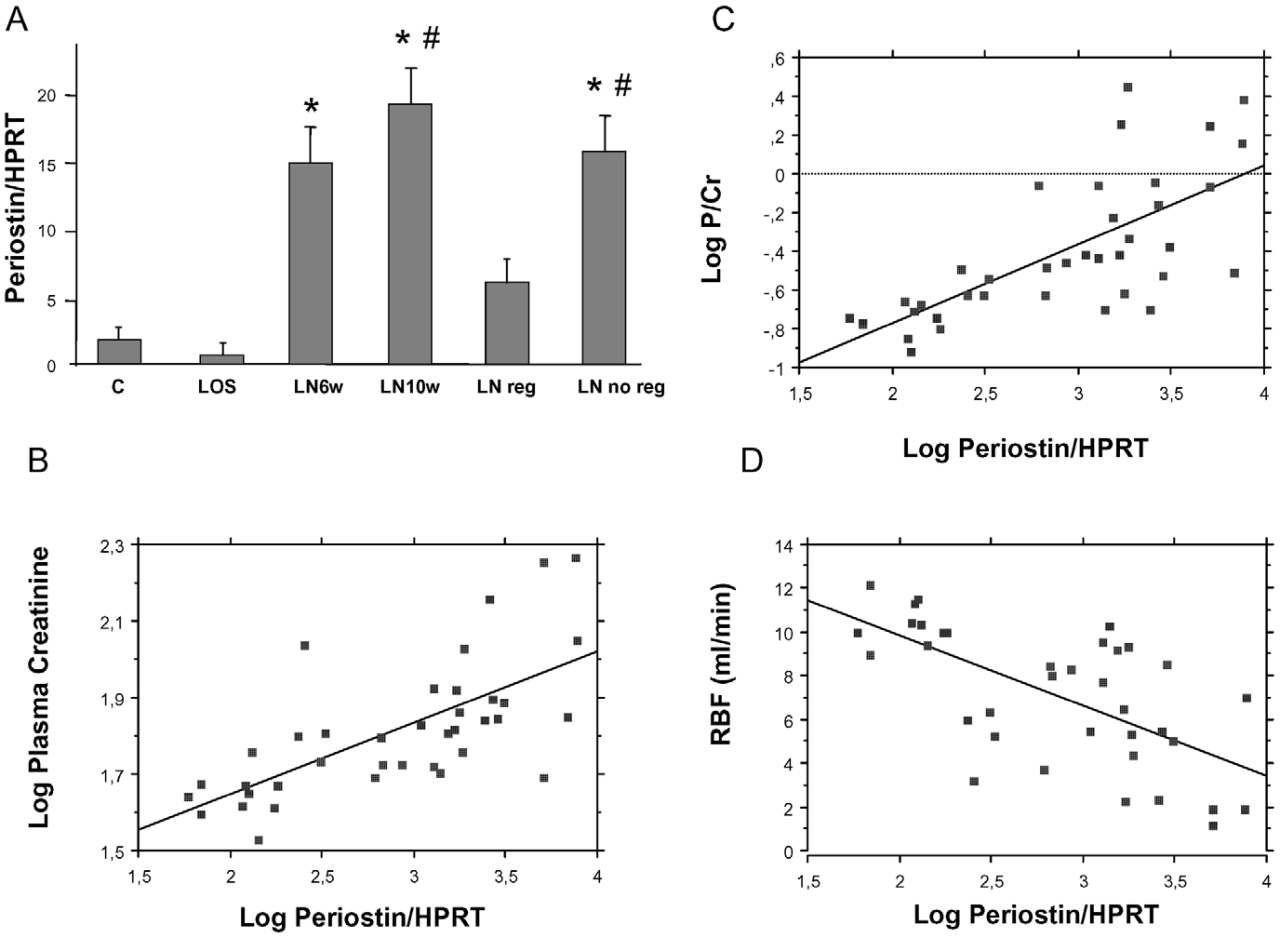
Periostin expression is correlated to the progression of renal disease. (A): Real-time quantitative PCR for periostin renal mRNA expression. Results are expressed as arbitrary units. Error bars represent SEM. * *p*<0.05 *vs* C ; # *p*<0.05 *vs* LN Reg. (B–D)Logarithmic regression between renal periostin mRNA and plasma creatinine, proteinuria/creatininuria, and renal blood flow.

N for each group is shown in Figure 3. Values are mean ± SEM.

* *p*<0.05 *vs* C:

# *p*<0.05 *vs* LN Reg.

### Losartan promotes regression of renal disease in a subpopulation of LNAME-treated rats

To study determinants of renal disease progression/regression, hypertensive rats displaying standardized mild nephropathy (proteinuria/creatininuria ∼1 g/mmol) were treated with losartan, together with the continuation of L-NAME. Important heterogeneity in the evolution of renal disease was observed.

Rats were thereafter separated in two groups according to the median value of plasma creatinine after 4 weeks losartan+L-NAME treatment (Figure S1). Accordingly, the “No Regression” (No Reg) group presented an escape of renal disease with a significantly higher plasma creatinine compared to the “Regression” (Reg) group, which exhibited reversal of renal disease (Table 1). Consistent with the differential therapeutic efficiency of losartan between the 2 groups, No Reg rats presented aggravated renal blood flow, vascular lesions, glomerulosclerosis and renal inflammation compared to Reg group (Figure 1, Table 1).

### Periostin is closely associated with the progression/reversal of experimental hypertensive nephropathy

Transciptomic analysis was performed in a limited number (n = 4) of animals from the No Reg and Reg groups comparing the expressions of 180 genes involved in fibrosis, extracellular matrix regulation and inflammation. Table S1 shows the genes that were significantly upregulated. Among them, periostin showed the highest overexpression and since it was not reported before to be involved in renal disease, we investigated in subsequent studies more precisely its expression.

As shown by RT-qPCR, periostin was expressed in the rat kidney at baseline and was 13- and 18-fold up-regulated after 6 and 10 weeks L-NAME, respectively (Figure 3A). Importantly periostin mRNA was significantly blunted in Reg group, whereas No Reg group presented a persistent increase in periostin expression in spite of 4 weeks losartan treatment (Figure 3A).

**Figure 4:**
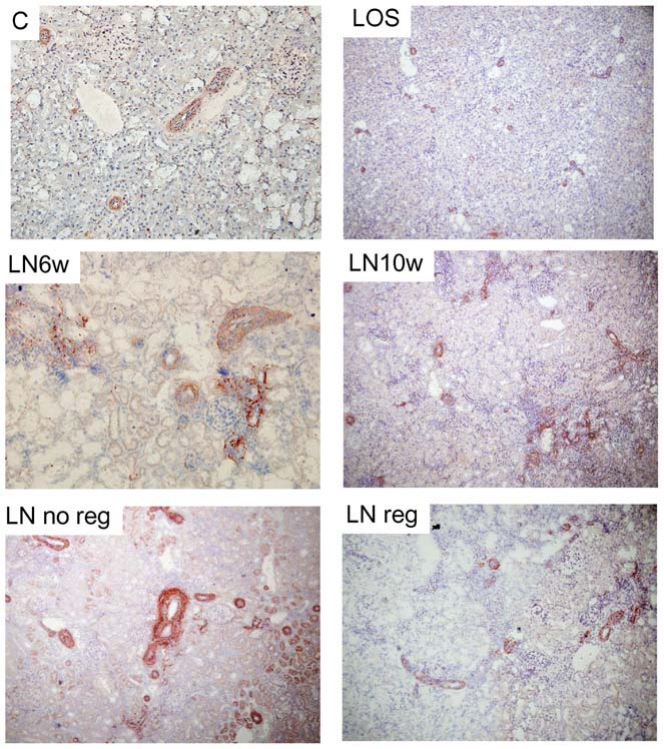
Expression of periostin by immunohistochemistry in renal cortex during L-NAME-induced hypertensive nephropathy. Original magnification ×20.

Immunostainings revealed a weak expression of periostin in the normal rat kidney, within the media of arteries and arterioles. Hypertensive nephropathy was characterized by a strong increase in periostin staining in the media and the adventitia of renal vessels. Interestingly periostin also exhibited a focal *de novo* interstitial expression in close vicinity to the most severe vascular, glomerular and tubular lesions (Figure 4).

To evaluate the importance of periostin as a marker and/or an actor of kidney disease progression, we performed regression analyses with hallmarks of hypertensive nephropathy severity as dependent variables. We found a very strong association between periostin mRNA expression and plasma creatinine (r = 0.68, *p*<0.001), proteinuria (r = 0.71, *p*<0.001) and renal blood flow (r = −0.64, *p*<0.001) (Figure 3B, Table 2). Periostin presented stronger associations with the latter dependent variables when compared to classical players of the proinflammatory and profibrotic process involved in hypertensive nephropathy (Table 2). Of note, when systolic blood pressure was added as an independent variable in multiple regression analyses the associations between periostin and creatinine (*p*<0.01), proteinuria (*p*<0.001) and renal blood flow (*p*<0.05) held true.

**Table 2:** Univariate regression analyses between kidney disease progression hallmarks (as dependent variables) and renal cortex mRNA expression of selected genes (as independent variables).

	Plasma Creatinine	Proteinuria/Creatininuria	Renal Blood Flow
Periostin	0.68***	0.71***	−0.64***
Col1A2	0.41**	0.57***	−0.45**
Col3A1	0.42**	0.36*	−0.41*
MMP2	0.52***	0.32	−0.32
Vimentin	0.63***	0.67***	−0.59***
MCP-1	0.22	0.22	−0.22
VCAM-1	0.38*	0.20	−0.30
ESel	0.40*	0.47**	−0.33*
ET-1	0.38*	0.51***	−0.61***

All variables were log-transformed, except from Renal Blood Flow which exhibited normal distribution.

Values are r coefficients.

* *p*<0.05;

** *p*<0.01;

*** *p*<0.001.

### In contrast with periostin, markers of endothelial dysfunction, epithelial mesenchymal transition or fibrillar collagen synthesis did not discriminate between regressive or not regressive experimental nephropathy

**Figure 5:**
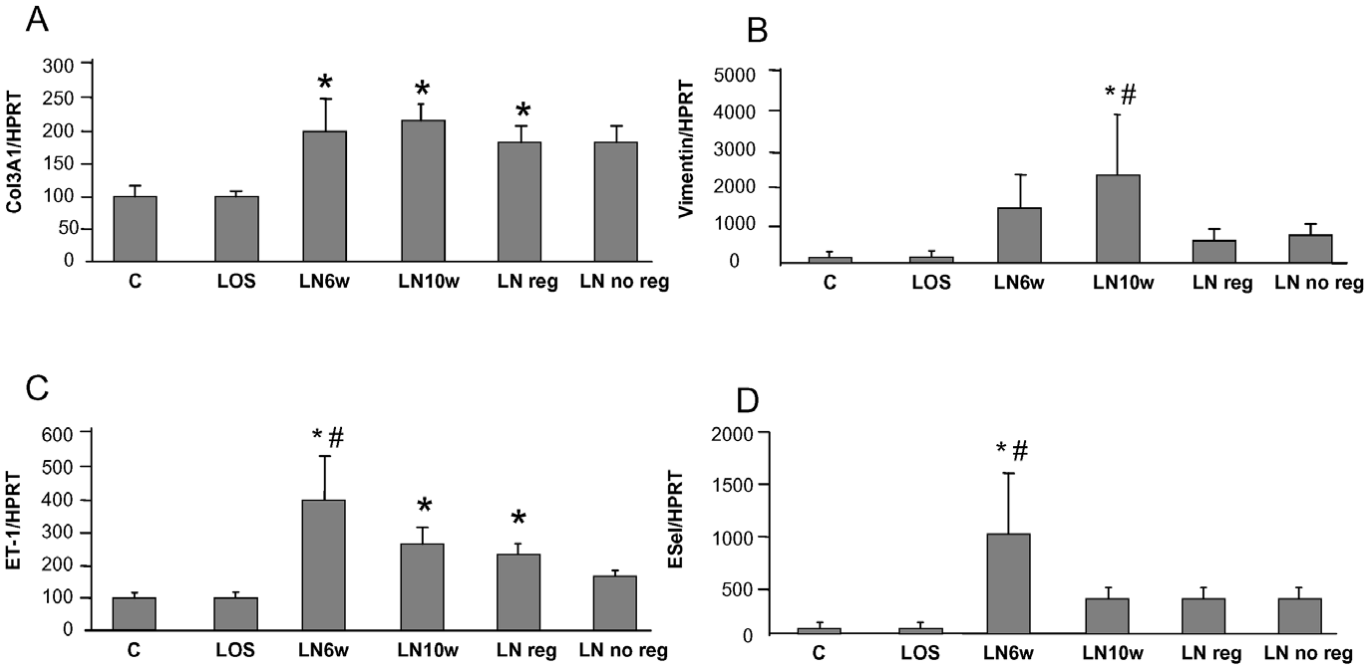
Markers of endothelial dysfunction, epithelial mesenchymal transition and fibrosis during progression/regression of renal disease. Real-time quantitative PCR for endothelin-1 (ET1, A) and E-selectin (ESel, B) Col3A1 (C) and vimentin (D). Results are expressed as arbitrary units. Error bars represent SEM. * *p*<0.05 *vs* C ; # *p*<0.05 *vs* LN Reg.

Since endothelial injury is present in hypertension and may play a central role in the pathophysiology of renal vascular diseases, we investigated the transcriptional regulation of ET-1 propeptide and E-selectin in this model (Figure 5A and 5B). Interestingly, both markers presented a strong but transient induction at week 6, suggesting early endothelial dysfunction. However, neither E-selectin, nor ET-1 enabled to discriminate Reg group from No Reg group during the progression of the disease.

Although significant fibrosis was detected on week 10, but not on week 6 (Table 1), Col3A1 RT-qPCR was already up-regulated on week 6, indicating persistently increased synthesis of collagen from this stage onwards (Figure 5C). Note that Col3A1 synthesis was not different between Reg and No Reg groups. Vimentin was progressively induced at week 6 and week 10 (Figure 5D). In contrast to Col3A1, vimentin exhibited a sharp decrease after the initiation of losartan, irrespective of the Reg/No Reg group.

### Periostin is overexpressed in human chronic renal allograft

**Figure 6:**
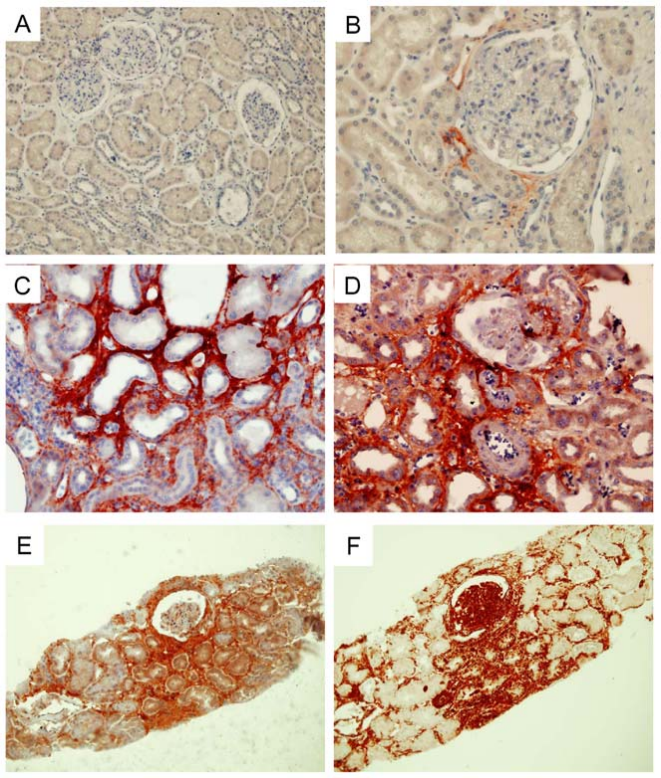
Periostin immunohistochemistry in normal human kidney (A, B), and chronic allograft nephropathy (C, D). Original magnification ×40. Periostin (E) and vimentin (F) staining on serial sections in chronic allograft nephropathy. Original magnification ×20.

To determine whether the latter findings may translate to human disease, we further analyzed periostin immunostaining on human kidney biopsy specimens. Periostin was not expressed in glomeruli or tubules in normal renal tissue. Only a weak staining could be found in small vessels (Figure 6A, 6B). Chronic allograft nephropathy is a clinical condition characterized by tubulointerstitial inflammation and fibrosis. In this condition we observed an intense periostin expression, predominantly in the region presenting tubular atrophy and interstitial fibrosis, as well as in several tubular epithelial cells (Figure 6C, 6D). Using serial sections with periostin and vimentin, the latter indicating epithelial phenotypic changes associated with renal graft fibrosis progression, the expression of periostin was mainly located in the interstitial area around the injured tubules, suggesting the importance of periostin expression during human kidney injury (Figure 6E, 6F).

## Discussion

The renin-angiotensin system is a central player in multiple mechanisms responsible for the progression of renal fibrosis [11]–[13]. Timely blockade with ACE inhibitors, renin inhibitors or AT1 receptor antagonists can promote reversal of renal lesions and ultimately prevent the evolution towards end-stage organ failure in experimental models of renal disease [9], [10], [14]. However, renin-angiotensin system blockers are inconstantly efficient in preventing chronic renal disease progression, particularly in humans, although the factors involved in this variable efficacy remain unclear [1], [15].

In this study we analyzed the reversal of L-NAME hypertensive nephropathy with losartan, and focused on the identification of markers of a non-return point of renal disease reversal. Our results show that losartan ameliorated renal hemodynamic alterations, proteinuria, and vimentin expression in all groups treated, compared to L-NAME 6w and 10w groups. Since these variables are classical determinants of renal disease progression, it could have been expected that the latter effects would be associated with a global protection against the functional and structural alterations ultimately induced by NO-deficiency hypertension. Instead, in spite of these beneficial effects, the No Reg group presented severe functional and structural disease, characterized by elevated plasma creatinine and vascular fibrosis similar to LN 10w group. Important additional factors implicated in renal disease progression and influenced by losartan are therefore responsible for the differential evolution between the Reg and No Reg groups. The reason why these important factors were different between the two groups is uncertain. In spite of the standardized cut-off point chosen to introduce losartan, we cannot exclude that the heterogeneity observed between Reg and No Reg groups may be due to subtle differences in the evolution of the renal disease around the non-return point, when losartan was introduced. Alternatively, preexisting heterogeneity in the regulation of pro- or anti-fibrotic genes between the Sprague Dawley rats, an outbred strain in which genome is not totally identical between animals, could account for the differences between the Reg and No Reg groups. We took advantage of these original experimental conditions to perform a transcriptomic analysis of selected markers associated with the progression of hypertensive nephropathy.

In the Reg group, rats presented reduced renal fibrosis compared to the No Reg group. Renal fibrosis is characterized by the accumulation of extracellular matrix, including fibrillar collagen. Since collagen III production, evaluated by Col3A1 RT-qPCR, was not different between the Reg and No Reg groups, the histological differences observed may be due to increased degradation of fibrillar collagen, as previously described in our laboratory [9].

L-NAME hypertension is associated with features of endothelial dysfunction [16]–[18]. Our results show early endothelial activation indicated by increased expression of ET-1 propeptide and E-selectin in LN 6w group. We and others have previously identified ET-1, a potent profibrotic vasoconstrictor, as a mediator of renal fibrosis in hypertensive nephropathy [10], [19]. E-selectin is an endothelial inducible adhesion molecule notably involved in the pathophysiology of atherosclerosis and in renal ischemia-reperfusion, but its potential implication in hypertensive nephropathy has not been reported previously. Although the present study shows early increase in renal ET-1 and E-selectin mRNA, their similar expressions in Reg and No Reg groups suggest that these markers of endothelial activation are not major determinants of the reversal of renal disease induced by AT1 receptor blockade. Similarly, vimentin, a classical marker of mesenchymal cells associated with fibrogenesis, was reduced by losartan treatment, irrespective of the reversal of renal disease.

Periostin is an extracellular matrix protein first identified in the periosteum and the periodontal ligament [20]. Angiotensin II can induce periostin expression in fibroblasts and vascular smooth muscle cells, via Ras/p38 MAPK/CREB and ERK1/2/TGF-beta1 pathways and via phosphatidylinositol-3-kinase signaling, respectively [21], [22]. Accordingly, periostin is induced in models of ischemic, hypertensive and hypertrophic cardiomyopathies, and an AT1 receptor antagonist decreases the cardiac expression of periostin [23]–[25]. In the kidney, experimental studies evaluating the implication of periostin in physiology and disease are scarce. Periostin is transiently expressed during renal development [26], and the expression in the normal adult kidney is low. Recent studies observed overexpression of periostin in animal models of diabetic, renal subtotal ablation and ureteral obstruction nephropathies [27]. In humans, periostin staining was observed in biopsies of glomerular diseases and within the cysts in autosomal dominant polycystic kidney disease [28], [29]. In addition, increased urinary excretion rates of periostin were reported in a limited number of proteinuric and non-proteinuric CKD patients [27]. Whether periostin is implicated in hypertensive nephropathy and in the progression/reversal of chronic kidney disease remained unknown.

Our results identified a progressive induction of periostin in the kidney with the progression of the hypertensive nephropathy. Regression analyses found a strong association between periostin mRNA expression and robust functional markers of kidney disease. Importantly, these associations held true when systolic blood pressure was added as a covariate, which suggests that periostin is correlated to renal injury independently of the degree of hypertension. Immunohistochemical analyses revealed that the localization of periostin was predominantly perivascular, in areas where important deposits of extracellular matrix occur in this model. We also observed an intense predominantly extracellular staining for periostin in the injured fibrotic tubulo-interstitial regions of chronic allograft nephropathy, which further demonstrates overexpression of periostin in human kidney disease. Identification of the main cells responsible for the interstitial accumulation of periostin requires further investigation. Data from previous studies suggests that fibroblasts, smooth muscle cells and tubular epithelial cells may be involved in periostin expression in this setting [21], [22], [30].

We found that after the onset of hypertensive renal disease, curative treatment with losartan was associated with diminished periostin expression in Reg, not in No Reg group, which suggests that the reduction of periostin may be implicated in the mechanisms of angiotensin II-related disease progression and that reduction of periostin expression may be a critical determinant of disease reversal. Interestingly, the analysis of histological fibrosis scores shows that the difference between Reg and No Reg groups was most evident for perivascular fibrosis, in accordance with periostin distribution in experimental hypertensive nephropathy. These original data suggest that periostin may be related to the pathophysiology of extracellular matrix accumulation at the site of renal injury.

Together, the results of this work identify periostin as a previously unrecognized marker associated with hypertensive nephropathy. Further research is necessary to precise the potential of renal, plasma and urine periostin as prognostic biomarkers to monitor the progression and therapeutic control of human chronic kidney diseases.

## Materials and Methods

### Animal treatment

Male Sprague-Dawley rats, weighing 250 g, were maintained on a normal-salt diet and had free access to chow and tap water. NO synthesis was inhibited by L-NAME (orally, 15 mg.kg^−1^.day^−1^). We have previously found that this dose produced a gradual elevation of blood pressure accompanied by the progression of renal disease. As shown in Figure S1, when proteinuria exceeded 1 g/mmol creatinine (∼6 weeks), a group of animals was killed to allow estimations of renal hemodynamics and morphological parameters just before the beginning of therapy (LN 6w group, n = 10). The remaining animals were divided into two subgroups for an additional experimental period of 4 weeks: in the first subgroup, L-NAME was given alone (LN 10w group, n = 12); in the second subgroup treatment was accompanied by the oral administration of an AT1 receptor antagonist (losartan, 30 mg.kg^−1^.day^−1^ in drinking water, Merck Sharp and Dohme-Chibret). At the end of losartan treatment, we separated the animals of the second group in two subgroups: animals with creatininemia below (LN Reg, n = 14), and above or equal to the median value (LN No Reg, n = 15). The doses of the drugs were based on pilot experiments and previously published studies [8], [30]. Control animals were sacrificed at 6 and 10 weeks. Since control animals presented similar results for all measured parameters, pooled data are presented (C group, n = 8). Three animals received losartan during 4 weeks as additional control group (LOS group, n = 3). All protocols and treatments were performed with the approval of the French government ethics committee.

### Renal hemodynamics

After anesthesia by pentobarbital sodium (50–60 mg/kg intraperitoneally, Nembutal, Abbott, Chicago, IL), animals were placed on a servo-controlled table kept at 37°C and the trachea was cannulated to facilitate respiration. The left femoral artery was catheterized for measurement of invasive arterial pressure, and a femoral venous catheter was used for infusion of volume replacement. An ultrasound transit-time flow probe (1RB, Transonic, Ithaca, NY) was placed around the left renal artery. Bovine serum albumin (4.7 g/dl of saline solution) was infused initially at 50 µl/min to replace surgical losses, and then at 10 µl/min for maintenance. Arterial pressure was measured via a pressure transducer (Statham P23 DB); RBF was measured by a flowmeter (T 420, low-pass filter, 40 Hz, Transonic). RBF values were controlled for zero offset determined at the end of an experiment after cardiac arrest. Data were recorded, stored, and analyzed using a DataTranslation analog-to-digital converter and the IOX software (EMKA Technologies, Paris, France).

### Urinary protein excretion and plasma creatinine

Morning urine samples were collected over a standardized 4-h period. Urinary protein concentration was normalized to creatinine concentration, and values were expressed as g protein per mmol creatinine. Central venous blood samples were withdrawn on the last day of the study, and plasma creatinine (µmol/l) was measured by automated Jaffe's method.

### Renal histology

Kidneys were stained with Masson's trichrome solution. Four-µm sections of kidneys were examined on a blinded basis by two investigators independently to estimate inflammation, tubular lesions, interstitial fibrosis, vascular fibrosis, glomerular sclerosis and vascular necrosis using a 0 to 4 injury scale as described previously [7], [8]. Lesion indexes from individual sections were averaged to calculate a sclerotic index for each rat.

### Immunohistochemistry for CD3

Four-micrometer-thick sections of paraffin-embedded kidneys were dewaxed, heated in citric acid solution (pH 6) at 98°C for 30 min, and incubated first with a polyclonal goat anti-rat CD3 antibody recognizing T lymphocytes (Santa Cruz Biotechnology, Santa Cruz, CA) for two hours at 37°C and then incubated for 30 min at room temperature with a second antibody from N-Histofine kit (Nichirei Biochemicals, Japan). Staining was revealed by applying AEC (Dako), counterstained with hematoxylin QS (Vector, Burlingame, CA), and finalized with Permanent Aqueous Mounting Media (Innovex). Quantification of CD3-positive cells was performed using Olympus analysis software.

### Immunohistochemistry for periostin and vimentin

On rat tissue, 4-µm thick frozen sections were incubated with polyclonal rabbit anti-rat periostin antibody (Abcam, Cambridge, UK) overnight at 4°C and then incubated for 30 min at room temperature with a second antibody from N-Histofine kit (Nichirei Biochemicals, Japan). Staining was revealed by applying AEC (Dako), counterstained with hematoxylin QS (Vector, Burlingame, CA), and finalized with Permanent Aqueous Mounting Media (Innovex).

Renal biopsies from patients (1 normal kidney, 1 chronic allograft nephropathy) were retrospectively analyzed. Informed consent was given for use of part of the biopsy for scientific purpose. All procedures and use of tissue were performed according to the national ethical guidelines and were in accordance with the declaration of Helsinki. Paraffin-embedded sections were dewaxed and hydrated. The antigens were retrieved by 20-min boiling in 10 mM citric acid solution (pH 6). The sections were incubated overnight with 1/4000 anti-periostin polyclonal rabbit antibody (Biovendor, France) or 1/1000 anti-vimentin mouse monoclonal antibody V9 (Zymed, Invitrogen, Cergy Pontoise, France). The sections were then incubated with anti-rabbit or anti-mouse antibody conjugated with peroxydase-labeled polymer (Dako, Trappes, France). Immunoreactive proteins were visualized with a 3-amino-9-ethylcarbazole-containing peroxydase kit (Dako) and counterstained with hematoxylin. For negative controls, the primary antibody was replaced by an equal concentration of rabbit or mouse IgG.

### Real Time quantitative PCR and Transcriptomic Analysis

We extracted RNA from the renal cortex and the abdominal aorta using TRIzol solution (Life Technologies BRL, Gaithersburg, MD). RNA quality was checked by measuring the ratio of optical densities at 260 and 280 nm and residual genomic DNA was removed by DNase I treatment for 30 min at 37°C (Fermentas). We used reverse transcription with Revert Aid H minus First Strand DNA Synthesis kit (Fermentas) to convert 1 µg RNA into cDNA. Transcriptomic analyses were performed with RT^2^Profiler PCR Array (Superarray, Bioscience Corp, Tebu Bio, Le Perray en Yvelines, France). cDNA was amplified by PCR using a LightCycler 480 (Roche Diagnostic) using SYBR Green (Fast Start DNA Master SYBRGreen I; Roche Applied Science, Roche Diagnostic), specific primers for selected mRNA and hypoxanthine-guanine phosphoribosyltransferase (HPRT) as housekeeping gene under the following conditions: 95°C for 5 min, and 45 cycles at 95°C for 15 s and 60°C for 15 s, then 72°C for 15 s. Specific primers were designed by Universal Probe Library system (UPL, Roche Applied Science), sequences are shown in Table 3. To normalize the qRT-PCR results we used Roche LightCycler 2.0 software (Roche Diagnostics). We expressed results as 2^−ΔCp^, where Cp is the cycle threshold number. We analyzed dissociation curves after each run for every amplicon to assess the specificity of quantification when using SYBR Green.

**Table 3:** Primers used for qRT-PCR.

mRNA	Strand	Sequence
Periostin	Sense	5′- TCGTGGAACCAAAAATTAAAGTC - 3′
	Antisense	5′- CTTCGTCATTGCAGGTCCTT - 3′
Col3A1	Sense	5′- CAAGGCTGAAGGAAATAGCAA - 3′
	Antisense	5′- TGCTCCATTCACCAGTGTGT - 3′
Col1A2	Sense	5′- CCTGGCTCTCGAGGTGAAC - 3′
	Antisense	5′- CAATGCCCAGAGGACCAG - 3′
MCP-1	Sense	5′- AGCATCCACGTGCTGTCTC - 3′
	Antisense	5′- CACCCACAGTGGACATAGCA - 3′
VCAM-1	Sense	5′- CAAATGGAGTCTGAACCCAAA - 3′
	Antisense	5′- GGTTCTTTCGGAGCAACG - 3′
MMP2	Sense	5′- GCACCACCGAGGATTATGAC - 3′
	Antisense	5′- CACCCACAGTGGACATAGCA - 3′
ESel	Sense	5′- AGGCTTCAGTGTGGTCCAA - 3′
	Antisense	5′- CAAGGCTTGAACACTGTACCC - 3′
Vimentin	Sense	5′- TTCTTCCCTGAACCTGAGAGA - 3′
	Antisense	5′- GAGTGGGTGTCAACCAGAGG- 3′
TGF-beta1	Sense	5′- CCTGGAAAGGGCTCAACAC - 3′
	Antisense	5′- CAGTTCTTCTCTGTGGAGCTGA - 3′
ET-1	Sense	5′- TGTCTACTTCTGCCACCTGGA - 3′
	Antisense	5′- CCTAGTCCATACGGGACGAC - 3′
HPRT	Sense	5′- GACCGGTTCTGTCATGTCG - 3′
	Antisense	5′- ACCTGGTTCATCATCACTAATCAC - 3′

### Statistics

Statistical analyses for the *in vivo* studies were performed using ANOVA followed by Fisher's protected least significance difference test in the Statview software package. All values are means ± SEM. Univariate and multivariate regression analyses between selected mRNA qRT-PCR values and quantitative surrogate markers for kidney disease progression were computed after log transformation for non-normally distributed variables. Results with *p*<0.05 were considered statistically significant.

## Supporting Information

Figure S1 **Experimental protocol. P/C: proteinuria/creatininuria.**
(PPT)

Table S1 **Listing of genes overexpressed in the animals escaping the losartan treatment (LN No Reg) compared to the animals responding to the losartan treatment (LN Reg).**
(PPT)
