# Supplementary material for: Identification of Periostin as a Critical Marker of Progression/Reversal of Hypertensive Nephropathy
Source: PLoS One. 2012 Mar 5;7(3):e31974. doi: 10.1371/journal.pone.0031974 (PMC3293874; doi:10.1371/journal.pone.0031974)
Supplement: Figure S1 — Experimental protocol. P/C: proteinuria/creatininuria. (PPT) [file pone.0031974.s001.ppt]

## Slide 1
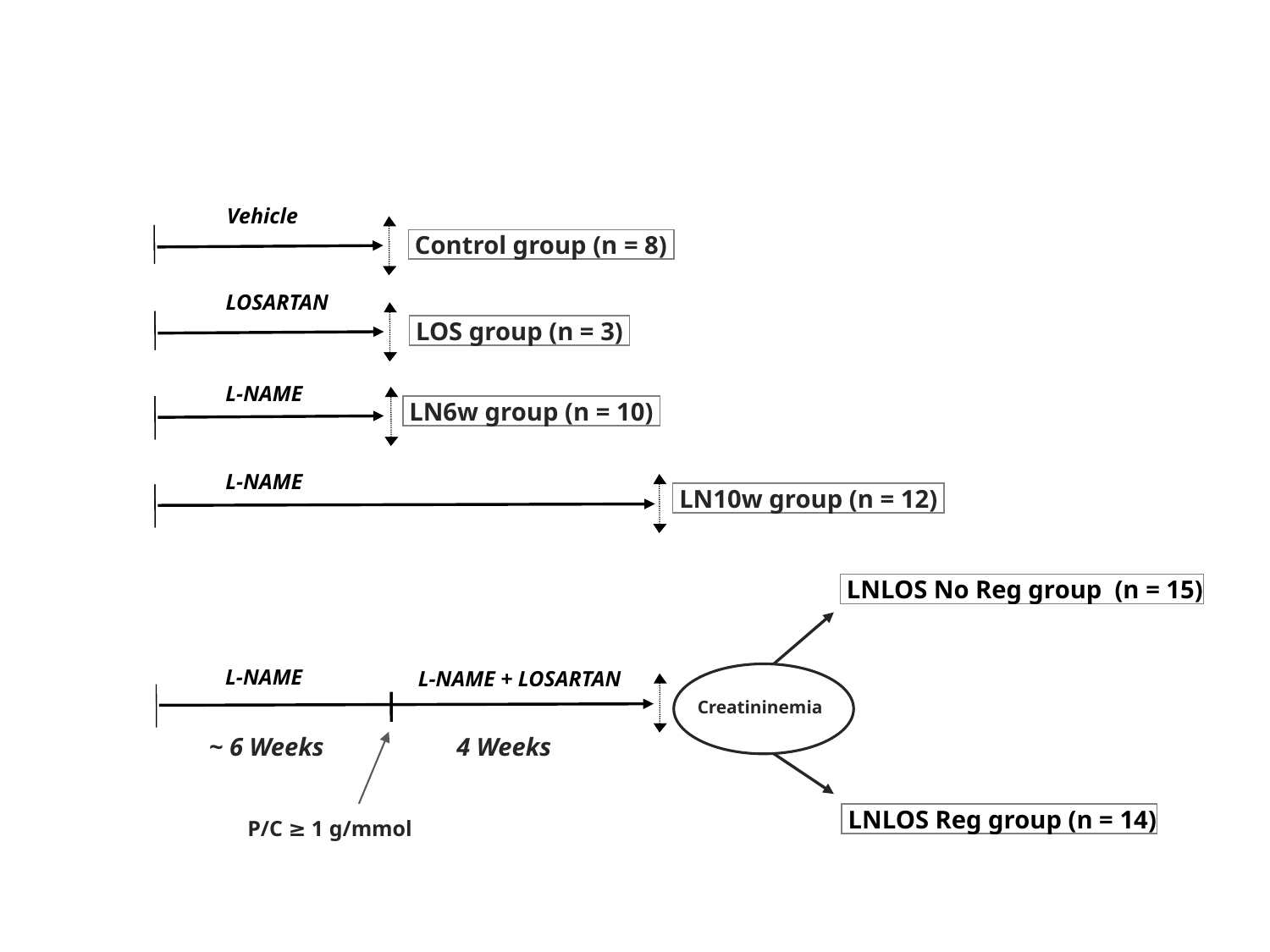

Vehicle
 Control group (n = 8)
LOSARTAN
 LOS group (n = 3)
L-NAME
 LN6w group (n = 10)
L-NAME
 LN10w group (n = 12)
 LNLOS No Reg group (n = 15)
L-NAME
L-NAME + LOSARTAN
Creatininemia
~ 6 Weeks
4 Weeks
 LNLOS Reg group (n = 14)
P/C ≥ 1 g/mmol
