# Supplementary figures and images for: Identification of Periostin as a Critical Marker of Progression/Reversal of Hypertensive Nephropathy
Source: PLoS One. 2012 Mar 5;7(3):e31974. doi: 10.1371/journal.pone.0031974 (PMC3293874; doi:10.1371/journal.pone.0031974)

## Slide 1
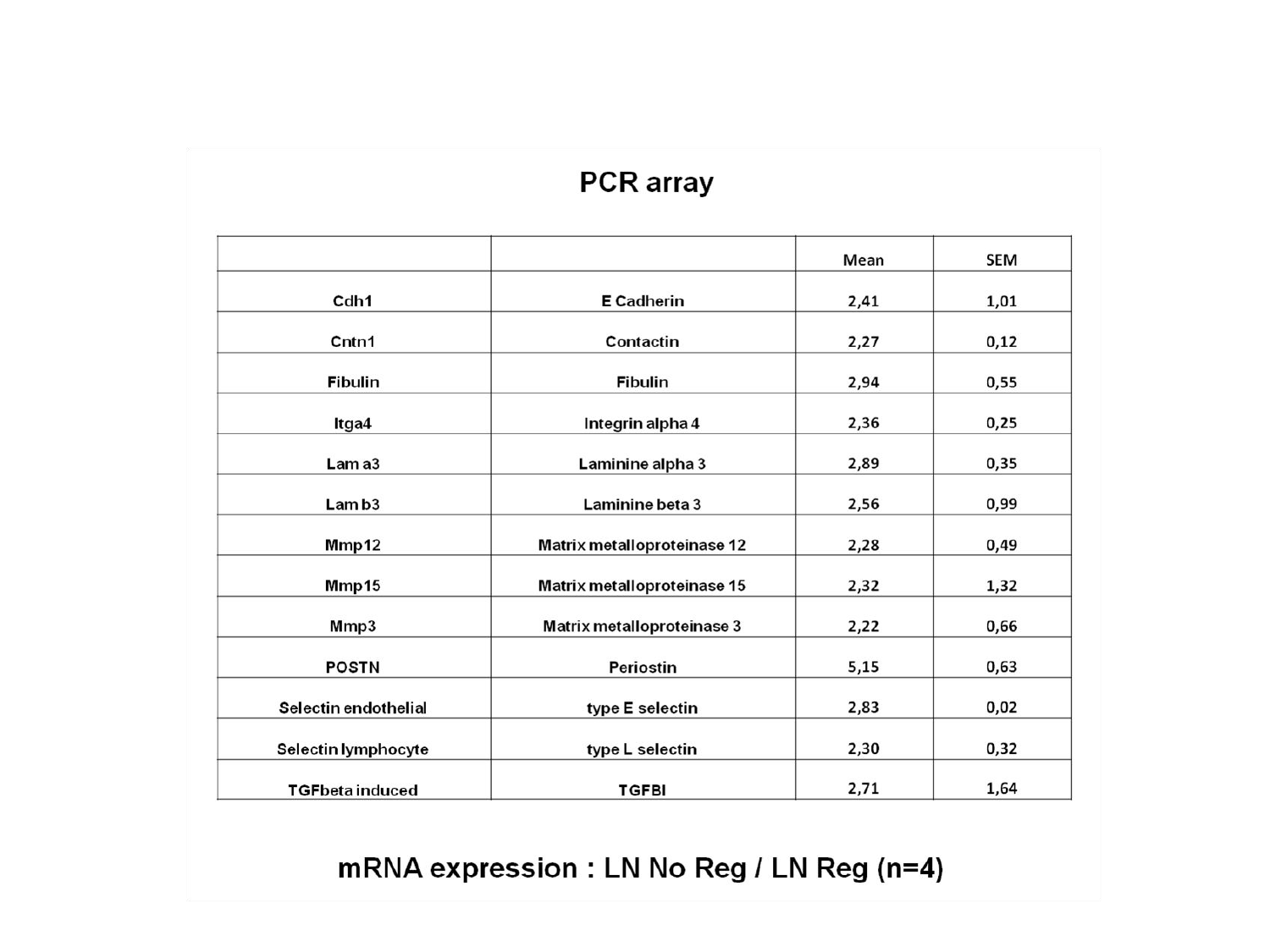

Supplement: Table S1 — Listing of genes overexpressed in the animals escaping the losartan treatment (LN No Reg) compared to the animals responding to the losartan treatment (LN Reg). (PPT) [file pone.0031974.s002.ppt]
